# *Leishmania amazonensis* Amastigotes Highly Express a Tryparedoxin Peroxidase Isoform That Increases Parasite Resistance to Macrophage Antimicrobial Defenses and Fosters Parasite Virulence

**DOI:** 10.1371/journal.pntd.0003000

**Published:** 2014-07-17

**Authors:** Calvin A. Henard, Eric D. Carlsen, Christie Hay, Peter E. Kima, Lynn Soong

**Affiliations:** 1 Department of Microbiology and Immunology, University of Texas Medical Branch, Galveston, Texas, United States of America; 2 Institute for Human Infections and Immunity, University of Texas Medical Branch, Galveston, Texas, United States of America; 3 M.D.-Ph.D. Combined Degree Program, University of Texas Medical Branch, Galveston, Texas, United States of America; 4 Department of Pathology, University of Texas Medical Branch, Galveston, Texas, United States of America; 5 Department of Microbiology and Cell Science, University of Florida, Gainesville, Florida, United States of America; University of Antwerp, Belgium

## Abstract

Professional phagocytes generate a myriad of antimicrobial molecules to kill invading microorganisms, of which nitrogen oxides are integral in controlling the obligate intracellular pathogen *Leishmania*. Although reactive nitrogen species produced by the inducible nitric oxide synthase (iNOS) can promote the clearance of intracellular parasites, some *Leishmania* species/stages are relatively resistant to iNOS-mediated antimicrobial activity. The underlying mechanism for this resistance remains largely uncharacterized. Here, we show that the amastigote form of *L. amazonensis* is hyper-resistant to the antimicrobial actions of cytokine-activated murine and human macrophages as compared to its promastigote counterpart. Amastigotes exhibit a marked ability to directly counter the cytotoxicity of peroxynitrite (ONOO^−^), a leishmanicidal oxidant that is generated during infection through the combined enzymatic activities of NADPH oxidase and iNOS. The enhanced antinitrosative defense of amastigotes correlates with the increased expression of a tryparedoxin peroxidase (TXNPx) isoform that is also upregulated in response to iNOS enzymatic activity within infected macrophages. Accordingly, ectopic over-expression of the TXNPx isoform by *L. amazonensis* promastigotes significantly enhances parasite resistance against ONOO^−^ cytotoxicity. Moreover, TXNPx-overexpressing parasites exhibit greater intra-macrophage survival, and increased parasite growth and lesion development in a murine model of leishmaniasis. Our investigations indicate that TXNPx isoforms contribute to *Leishmania's* ability to adapt to and antagonize the hostile microenvironment of cytokine-activated macrophages, and provide a mechanistic explanation for persistent infection in experimental and human leishmaniasis.

## Author Summary

Pathogens of the genus *Leishmania* are the causative agents of leishmaniasis, a neglected tropical disease responsible for significant morbidity and mortality worldwide. Although it is well accepted that host-derived leishmanicidal molecules mediate resolution of *Leishmania* infection, some *Leishmania* species/stages are relatively resistant to host cell antimicrobial activity. These intracellular pathogens have developed evasive strategies to subvert host antimicrobials, and promote pathogen survival within the harsh intracellular environment. However, the underlying mechanisms remain largely uncharacterized. Here, we show that *L. amazonensis*, an agent of persistent infection in humans and non-healing skin lesions in mice, antagonize macrophage antimicrobial activity. The superb ability of the amastigote form to survive within host cells is related to its increased expression of a tryparedoxin peroxidase isoform that confers resistance to the cytotoxicity of host-derived antimicrobial molecules. Parasites induce higher expression of the TXNPx in response to iNOS activity during infection of macrophages, indicating that parasites can “sense” the microenvironment of host cells and regulate the expression of relevant virulence factors accordingly. Our investigations are consistent with a model by which *Leishmania* amastigotes utilize TXNPx to defend against host-derived molecules thereby promoting their intracellular survival and persistent infection.

## Introduction

*Leishmania spp*. are the causative agent of the neglected tropical disease leishmaniasis, causing significant morbidity and mortality worldwide. Leishmaniasis presents with a broad spectrum of clinical symptoms, ranging from self-healing cutaneous lesions to life-threatening systemic disease that is dependent on a complex interaction between the infecting parasite species and the host immune responses. *Leishmania* exhibit a digenetic lifecycle, converting from a flagellated promastigote stage in the sand fly vector to a non-motile amastigote stage within vertebrate host macrophages (MΦs). Amastigotes that are responsible for the disease survive and replicate in the hostile intracellular environment of MΦs.

Oxygen-dependent antimicrobial defenses, encompassing both reactive oxygen and nitrogen species (ROS and RNS, respectively), are extensively studied innate host defense mechanisms used against invading microorganisms. Upon phagocytosis of pathogens, MΦs generate superoxide (O_2_^•−^) via the NADPH phagocyte oxidase respiratory complex. O_2_^•−^ is subsequently dismutated enzymatically or spontaneously to hydrogen peroxide (H_2_O_2_), an oxidant that readily diffuses across membranes and has the propensity to generate the highly reactive hydroxyl radical (OH^•−^) through its reaction with iron. [1]. In addition, the diatomic radical NO, which is generated by iNOS during innate recognition of pathogen-associated molecular patterns or in the IFN-γ-primed response, is an intrinsic component of host defense to infection [2]. It is likely that the anti-*Leishmania* activity emanating from iNOS involves a myriad of NO congeners, including nitrogen dioxide (NO_2_), dinitrogen dioxide (N_2_O_3_), and peroxynitrite (ONOO^−^), which are generated in the reaction of NO with O_2_ and O_2_^•−^. In particular, the strong oxidant ONOO^−^ has been shown to mediate *Leishmania* toxicity [3], most likely through the nitration/and or oxidation of parasite membrane proteins [4]. These molecules are crucial for controlling the parasite burden during the course of cutaneous and visceral leishmaniasis [5]–[7]. It is also known that both ROS and RNS can mediate cytotoxicity through the oxidation or S-nitrosylation of redox-active cysteine thiols in proteins essential for cell function [8], [9], and that the leishmanicidal effect of NO can partially be attributed to inactivation of the cysteine proteinase virulence factor through S-nitrosylation of the Cys^25^ catalytic residue [10], [11].

In order to survive in the harsh phagolysosomal compartment of MΦs, *Leishmania* have developed a distinct system for defense against host-derived reactive species. These parasites are known to actively decrease production of ROS/RNS by interfering with NADPH oxidase assembly [12]–[14], or through the disruption of signaling pathways governing iNOS transcription [15]. They also can enzymatically detoxify reactive species generated endogenously during parasite metabolism, or exogenously via the anti-parasite immune response in the sandfly midgut or within professional phagocytes. *Leishmania* possess several antioxidant systems, including superoxide dismutases, peroxidases, and low-molecular weight thiols [16]. In contrast to higher eukaryotes, *Leishmania* do not express catalases or selenium-containing glutathione peroxidases; instead, they rely on the dithiol trypanothione to maintain their redox homeostasis. Trypanothione transfers reducing equivalents to the downstream antioxidant enzyme couple tryparedoxin (TXN)/tryparedoxin peroxidase (TXNPx) [17], forming a key antioxidant cascade vital for *Leishmania* resistance to ROS/RNS [18], [19]. The TXNPx enzymes are two-cysteine peroxiredoxins that act as homodimers to detoxify H_2_O_2_, organic hydroperoxides, ONOO^−^, and NO, and are highly conserved among the kinetoplastids [20]–[22]. *Leishmania* encode at least two TXNPx isoforms: one cytosolic form (a 199-amino acid protein with a predicted molecular mass of 20.1 kDa) and a mitochondrial form (a 226-amino acid protein with a predicted mature molecular mass of 21.4 kDa) [23]. These enzymes can promote *Leishmania* virulence due to their antioxidant and chaperone activities, respectively [24], [25]. In addition, a third developmentally-regulated isoform that is preferentially expressed by the mammalian parasite stage, termed TXNPx1 or Pxn1, has been identified [21], [26]. Collectively, these trypanothione-dependent processes are essential for *Leishmania* survival [27], [28], and are implicated in the development of parasite drug resistance [29]. Therefore, these pathways are actively being investigated as potential therapeutic targets for treatment of leishmaniasis.

Interestingly, susceptibility to ROS and RNS is highly variable among *Leishmania* species and developmental stages, even though genomic data indicate that all sequenced species possess similar antioxidant systems. For example, members of the *L. mexicana* complex (*L. mexicana*, *L. amazonensis*, *L. pifanoi*) exhibit enhanced resistance to direct treatment with oxyradicals and nitrogen oxides *in vitro* [30] and increased intracellular survival in cytokine-activated MΦs [31]. Parasites of the *L. mexicana* complex often cause persistent cutaneous lesions in humans and induce non-healing cutaneous lesions in BALB/c, C3H, and C57BL/6 strains of mice, suggesting that enhanced intra-MΦ survival is an important factor in host susceptibility. However, the mechanisms underlying the increased resistance of certain *Leishmania* species or stages to the antimicrobial actions of both oxyradicals and nitrogen oxides are unknown.

In this study, we compared the ability of *L. amazonensis* promastigotes and amastigotes to resist the antimicrobial activity of MΦs that were pre-activated with cytokines to boost their killing capacity. In addition, we evaluated the susceptibility of both parasite stages to authentic ROS and RNS, which are likely to be encountered by *Leishmania* in the parasitophorous vacuole. Our investigations indicate that while promastigotes are readily killed by cytokine-activated MΦs, axenic amastigotes are not only resistant to MΦ-mediate killing, but also grow intracellularly in spite of elevated host NO synthesis. In addition, amastigotes are more resistant to the leishmanicidal activity of RNS compared to promastigotes. Resistance of amastigotes to MΦ-mediated killing correlates with the increased expression of a TXNPx isoform by this stage of the parasite, (a 190-amino-acid protein with a predicted molecular mass of 20 kDa similar to *L. chagasi* Pxn1 [22]. Our findings uncover a novel *Leishmania* antioxidant/antinitrosative defense strategy that antagonizes iNOS enzymatic activity and fosters intracellular parasite fitness and virulence mechanisms. They also help explain why certain *Leishmania* species of the *L. mexicana* complex, including *L. amazonensis*, cause persistent disease in spite of Th1-like immune responses in infected or vaccinated hosts.

## Materials and Methods

### Ethics statement

Female wild-type C57BL/6 (B6), congenic iNOS-deficient (iNOS^−/−^) mice, and BALB/c mice were purchased from Taconic Laboratories (Hudson, NY). Mice were maintained under specific pathogen-free conditions and used at 6–10 weeks of age following protocols that have been approved by the Institutional Animal Care and Use Committee (protocol #9803016) at the University of Texas Medical Branch (UTMB) in Galveston, TX.

UTMB complies with the USDA Animal Welfare Act (Public Law 89-544), the Health Research Extension Act of 1985 (Public Law 99-158), the Public Health Service Policy on Humane Care and Use of Laboratory Animals, and the NAS Guide for the Care and Use of Laboratory Animals (ISBN-13). UTMB is a registered Research Facility under the Animal Welfare Act, and has a current assurance on file with the Office of Laboratory Animal Welfare, in compliance with NIH Policy.

### Mouse infections

C57BL/6 mice were inoculated s.c. with 5×10^6^ stationary-phase promastigotes in the right hind foot. Lesion development was evaluated biweekly for 9 weeks, at which time the mice were sacrificed for determination of parasite burden by using qRT-PCR as described below.

### Parasite cultures

Infectivity of *L. amazonensis* (RAT/BA/74/LV78) was maintained by regular passage through BALB/c mice. Promastigotes were cultured at 26°C in Schneider's medium (Invitrogen, Carlsbad, CA), pH 7.0, supplemented with 20% fetal bovine serum (FBS) (Hyclone, Logan, UT), and 50 µg/mL gentamicin (complete Schneider's medium). Axenic amastigotes were maintained at 32°C in Grace's medium (Invitrogen), pH 5.3 supplemented with 20% FBS. Promastigote growth in complete Schneider's medium was evaluated daily by direct counting of parasites using a hemacytometer. Metacyclic promastigote forms were purified using the 3A.1 monoclonal antibody, as previously described [32]. Tissue-derived amastigotes were harvested from foot tissues of infected BALB/c mice (∼12 wk post-infection) and cultured at 32°C for 48 h before use. To generate the TXNPx1-overexpressing strain, the TXNPx1 open-reading frame was amplified from *L. amazonensis* genomic DNA by using the forward 5′-AAAACCCGGGACCATGTCCTGCGGTGACGCCAA-3′ and reverse 5′-AAAACCCGGGTCACTTATTATGGTCGACCTTCAGGCCAGG-3′ primer set (*SmaI* restriction sites underlined), and directionally cloned into the *SmaI* site of pXG to generate pXG::TXNPx1. The orientation of the TXNPx1 open-reading frame was confirmed by PCR and sequencing. pXG and pXG::TXNPx1 were electroporated into logarithmic phase promastigotes by using the high-voltage (1500V) method, as previously described [33]. Stably transfected parasites were selected by growth in Schneider's medium containing G1418 (50 µg/mL). Confirmation of TXNPx1 overexpression was determined by immunoblotting. Promastigote and amastigote cultures carrying the episomal pSP72–YNEO-αIR-Luc1.2 [34] or pXG vectors were supplemented with G1418. Axenic amastigote or stationary promastigote cultures under ten passages were routinely used for *in vitro* infections.

### MΦ infections

Bone marrow-derived MΦs were generated from C57BL/6 and congenic iNOS^−/−^ mice, as previously described [35], and used for experimentation after 9 days of incubation with M-CSF (20 ng/mL). MΦs were seeded in 24-well culture plates (3×10^5^ cells/well) and allowed to attach overnight. The THP-1 monocytic cell line was activated with PMA (50 ng/mL) for 3 days prior to infection. Where indicated, MΦs and THP-1 cells were activated with LPS (100 ng/mL) and IFN-γ (100 U/mL) for 16 h prior to infection, which were maintained in the medium for the duration of the experiment; these cells are referred to as activated MΦs in all our experiments herein. The anti-leishmanial activity of MΦs and THP-1 cells was evaluated after challenge with a multiplicity of infection of 2 stationary-phase promastigotes or axenic amastigotes. After 1 h of infection, cells were washed with warm PBS to remove extracellular parasites, and new medium was added. Total RNA was harvested from infected MΦs, and cDNA was used in qPCR reactions with parasite-specific primers (forward 5′-AACGTGAACAACTGGATGTGCGTC-3′ and reverse 5′-ATGGTACCAAGCTTGACACATGCC-3′) directed against the single copy ubiquitin hydrolase (*Ubiq*) gene known to be constitutively expressed in both developmental stages [32]. Alternatively, genomic DNA was harvested from infected MΦs and used as a template in qPCR reactions with the *L. amazonensis* cysteine proteinase (*LaCys*)-specific primer set (forward 5′-TCGTGCTGGGCCTTCTC-3′ and reverse 5′-TTGCAGCCCACTGACCTT-3′). The relative parasite load was calculated by using the ΔΔCt method normalizing parasite *Ubiq* or *Cys* to MΦ *Gapdh* levels amplified with the murine specific forward 5′-GAGCTGAACGGGAAGCTCAC-3′ and reverse 5′-ACCACCCTGTTGCTGTAGC-3′ primers. Intracellular survival is expressed as the parasite burden determined at the indicated time relative to the parasite burden after 1 h of internalization. Nitrite in the MΦ culture supernatant was measured by the Griess reaction, and concentrations determined by regression analysis were compared to known nitrite standards. For secondary infections, amastigotes were released from infected cells by using 0.01% SDS in PBS, as previously described [36]. MΦ-derived amastigotes were counted, and equal parasite numbers (MOI 2) were then used to infect secondary activated MΦs. Parasite load was determined 48 h post-infection by qPCR.

### RNA isolation and quantitative RT PCR

Total RNA was isolated from lesions or 1×10^6^ infected MΦs by using the Qiagen RNeasy kit (Qiagen, Valencia, CA), and cDNA was synthesized by using 1 µg total RNA and iScript reverse transcriptase (Biorad, Hercules, CA). Real-time PCR was performed by using a BioRad iCyler iQ Real-Time PCR System (BioRad). The *TXNPx1* cDNA was amplified by using the forward 5′-ACCGCGGTCTCTTCATCATCGACCC-3′ and reverse 5′-TCACTTATTATGGTCGACCTTCAGGCCAGG-3′ primers. The cycle threshold (Ct) value for parasite *TXNPx1* or *Ubiq* mRNA was determined. Total parasite load in lesions was calculated using regression analysis based on *Ubiq* mRNA levels compared to a standard curve generated with amastigote-derived cDNA. Calculations were made considering 1 pg cDNA was equal to 7 parasites. Lesion *TXNPx1* mRNA levels were normalized to *Ubiq* mRNA levels to correct for parasite load, and *TXNPx1* expression levels in pXG control promastigote-infected lesions were set to 1.

### *In vitro* susceptibility to RNS and ROS

The leishmanicidal activities of the NO donor spermine NONOate (sNO, Cayman Chemical, Ann Arbor, MI), polyamine spermine (a negative control for sNO), hydrogen peroxide (H_2_O_2_), or peroxynitrite (ONOO*^−^*) were monitored by assessing luciferase activity in parasites that carry the episomal vector pSP72–YNEO-αIR-Luc1.2 [34]. sNO was assumed to exhibit a half-life of ∼100 minutes under our experimental conditions, based on its temperature- and pH-dependent NO release [37]. ONOO^−^ was freshly synthesized and quantified using UV-absorbance spectroscopy (ε_302 nm_ = 1670 M^−1^ cm^−1^), as previously described [38]. Since the parasitophorous vacuole is thought to be a nutrient limiting environment, we opted to perform the cytotoxicity assays in PBS in the absence of any exogenous metabolites. Neither parasite stage showed any loss of viability after 4 h of incubation in PBS alone (not shown). Bulk stationary-phase promastigotes or axenic amastigotes were diluted in PBS alone to a final concentration (1×10^6^ parasites/mL). For comparing susceptibility of promastigotes and amastigotes, 1×10^5^ parasites were aliquoted into 96-well plates and treated with the indicated concentrations of sNO, H_2_O_2_, or ONOO^−^ at 32°C for 4 h. Samples were lysed with luciferase assay buffer, and luciferase activity was determined using a luciferase assay system (Promega, Madison, WI) and measured as relative light units (RLU) on a Veritas microplate luminometer. The results are expressed as luciferase activity in treated samples relative to luciferase activity in untreated controls ×100%. For comparing susceptibility of transfected promastigotes, stably transfected stationary-phase promastigotes that carry either pXG or pXG::TXNPx1 were diluted in PBS (1×10^6^ parasites/mL), and 1×10^5^ parasites were seeded into 24-well plates and treated with the indicated concentrations of ONOO^−^ at 26°C for 4 h. After the treatment, 0.8 mL of complete Schneider's medium was added, and surviving parasites were cultured for 4 days. Parasites were enumerated by counting under a hemacytometer. Results are presented as parasites/mL in treated relative to untreated samples ×100%.

### Western blotting

Whole cell lysates were prepared from 1×10^8^ stationary-phase promastigotes, lesion-derived or axenic amastigotes, or 1×10^6^ infected MΦs. Samples normalized to 2 µg (parasite only lysate) or 10 µg (infected MΦ lysate) total protein were resolved by using 12% (v/v) SDS-PAGE, electrophoretically transferred to a nitrocellulose membrane, and immunoblotted with anti-TXNPx1 (generated by inoculating mice with the purified recombinant C-terminal 40 amino acids of the *L. pifanoi* truncated TXNPx1 isoform), anti-β actin (Sigma, A5441), anti-α tubulin (Sigma, T9026), and/or anti-luciferase (Sigma, L2164). α-tubulin was used as a loading control for comparing protein expression between promastigote samples. However, α-tubulin expression was relatively low in amastigotes (data not shown), presumably due to their lack of flagella, and anti-β actin was not a good loading control for lesion-derived amastigotes (data not shown). Therefore, we opted to use Ponsceau S staining as a loading control when comparing promastigote and amastigote protein expression levels. Band intensity was measured by using ImageJ software [39].

### Statistical analysis

One-way ANOVA was used for multiple group comparisons. Data from time course and titration experiments were evaluated by using a two-way ANOVA followed by a Bonferroni post-test. Differences between individual treatment groups were determined using a Student's t-test. A *p* value≤0.05 was considered statistically significant (GraphPad Software, San Diego, CA).

## Results

### Amastigotes resist the antimicrobial activity of cytokine-activated MΦs

MΦs readily phagocytose both promastigotes and amastigotes. However, our understanding of how the insect- or mammalian-stage of the parasite subverts the innate immune response of MΦs is incomplete. Most *in vitro* studies have used microscopic counting of parasites and host cells or conventional PCR assays to determine parasite burden, which have intrinsic limitations such as low sensitivity and high variation. For a more reproducible examination of *Leishmania* parasite survival and intracellular burden in control and cytokine-activated MΦs, we used qRT-PCR to quantify the amount of parasite RNA relative to host RNA in infected samples. In addition, luciferase-expressing promastigotes and amastigotes were used for some infections (Figure S1), allowing luciferase activity in infected MΦ lysates to be quantified and correlated to intracellular parasite loads. These molecular-based methods eliminate potential bias that exists with traditional microscopic counting methods commonly used to determine intracellular *Leishmania* load in *in vitro* infection studies.

**Figure 1:**
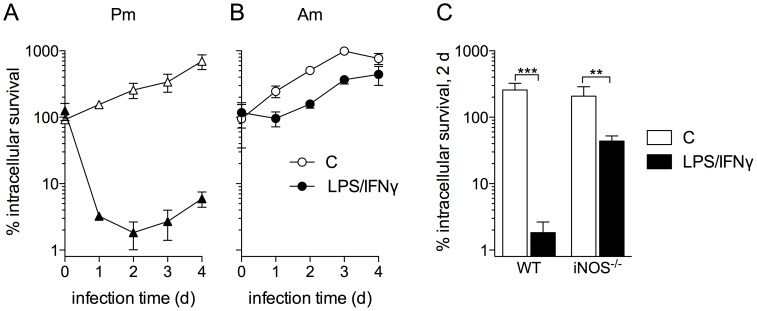
*L. amazonensis* amastigotes antagonize macrophage antimicrobial defenses. Survival of promastigotes (Pm, A) and amastigotes (Am, B) in control MΦs (white symbols/bars) and IFN-γ/LPS-activated MΦs (black symbols/bars). MΦs were activated with LPS (100 ng/mL) and IFN-γ (100 U/mL) for 16 h prior to infection. C) Pm survival in wild-type (WT) and iNOS-deficient (iNOS^−/−^) control and IFN-γ/LPS-activated MΦs at 2 days post-infection. A–C) Parasite loads determined by qRT-PCR normalizing the constitutively expressed parasite ubiquitin to MΦ Gapdh transcript levels. % survival was calculated comparing the parasite burden at the indicated time relative to the parasite burden after 1 h of internalization (t = 0). All infections were performed with an MOI of 2. The data represent the mean % survival ± SD of 4–8 independent observations from at least 2 separate experiments. ** *p*<0.01, *** *p*<0.001 compared to infected controls.

Upon infection of resting MΦs, both *L. amazonensis* promastigotes and amastigotes replicated efficiently with parasite loads increasing approximately 10-fold after 4 days of infection (Figures 1A and B, open symbols). However, we consistently found that ∼95% of ingested promastigotes were killed in IFN-γ/LPS-activated MΦs within 24 h of infection (Figure 1A and Figure S1A, closed symbols). The surviving parasite population began replicating in cytokine-activated MΦs at 48 h post-infection and continued to expand for the duration of the experiment (Figure 1A). In sharp contrast, amastigotes were resistant to MΦ microbicidal mechanisms (Figure 1B and Figure S1B, closed symbols). Amastigote replication in cytokine-activated MΦs was limited during the first 24 h of infection, but growth proceeded with similar kinetics in both control and activated cells thereafter (Figure 1B).

*Leishmania* are susceptible to the antimicrobial actions of RNS produced by the host iNOS hemoprotein [7], [40]. Therefore, we evaluated the ability of bone marrow-derived MΦs from C57BL/6 and iNOS^−/−^ mice to kill *L. amazonensis* promastigotes. In contrast to activated wild-type MΦs that efficiently killed ∼95% of parasites, primed iNOS^−/−^ MΦs only eliminated ∼50% of engulfed promastigotes (Figure 1C). This suggested that MΦ killing of promastigotes was largely dependent on iNOS; however, other MΦ effector mechanisms independent of NO clearly contribute to promastigote killing in the initial stages of infection when host cells are pre-activated [5]. Reportedly, *Leishmania* parasites can interfere with iNOS activity, thereby decreasing the effective NO concentration in infected cells [15]. To determine if the increased amastigote resistance to MΦ-mediated killing was due to a defect in RNS production, we measured the nitrite concentration in culture supernatants of infected cytokine-activated MΦs. Both promastigote- and amastigote-infected MΦs produced comparable levels of RNS at the time of infection (Figure S2A) and continued to produce similar concentrations of RNS up to 48 h post-infection (Figure S2B), suggesting that neither *L. amazonensis* promastigotes nor amastigotes interfere with iNOS production or activity in these primed cells.

Given that promastigote clearance in cytokine-activated murine MΦs was largely dependent on iNOS activity, and that human MΦs tend to produce less NO compared to murine cells [41], we evaluated the ability of primed human THP-1 monocytes to eliminate promastigotes and amastigotes. Similar to murine MΦs, luciferase-expressing amastigotes were resistant to THP-1 antimicrobial activity, while a significant percentage of internalized promastigotes were killed after 2 d of infection (Figure S1C, *p*<0.01). Together, these data indicate that *L. amazonensis* amastigotes are hyper-resistant to the antimicrobial activity of both murine and human MΦs and antagonize iNOS-mediated cytotoxicity in murine cells.

### Amastigotes exhibit increased resistance to nitroxidative stress compared to promastigotes

**Figure 2:**
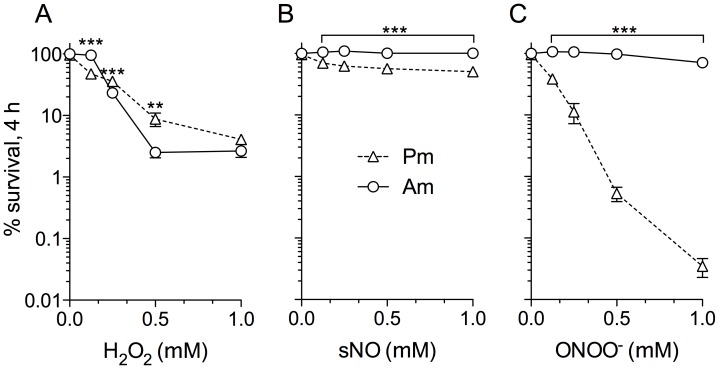
*L. amazonensis* amastigotes are hyper-resistant to oxyradicals and nitrogen oxides. Susceptibility of luciferase-expressing promastigotes (Pm) or amastigotes (Am) to increasing concentrations of A) hydrogen peroxide (H_2_O_2_), B) an NO releaser spermine NONOate (sNO), or C) peroxynitrite (ONOO^−^) in phosphate-buffered saline for 4 h. Percent survival was calculated by comparing luciferase activity in treated vs. untreated parasites.

Since much of the anti-*Leishmania* activity of cytokine-activated MΦs was dependent on iNOS, and amastigotes exhibited a survival advantage over promastigotes in these primed cells, we compared the leishmanicidal potential of H_2_O_2_, NO, and ONOO^−^, cytotoxic molecules produced during the innate MΦ response to *Leishmania*, against both parasite stages *in vitro*. Luciferase-expressing promastigotes and amastigotes were treated directly with increasing concentrations of H_2_O_2_, NO, and ONOO^−^, and luciferase activity at 4 h post-treatment was determined and used as a readout of parasite viability. Amastigotes were significantly more resistant than promastigotes after 4 h of exposure to 125 µM H_2_O_2_ (*p*>0.001); however, amastigotes became susceptible to higher H_2_O_2_ concentrations (250 µM-1 mM) (Figure 2A). Promastigotes exhibited a dose-dependent hyper-susceptibility to the NO-donor spermine NONOate (sNO), with 50% of parasites surviving 4 h after treatment with 1 mM sNO compared to untreated controls (Figure 2B, open triangles). In contrast, 100% of amastigotes survived 1 mM sNO (Figure 2B, open circles). Amastigotes were remarkably resistant to the strong oxidant ONOO^−^, which was in contrast to the extreme sensitivity of promastigotes (Figure 2C). At 0.5 mM concentration of ONOO^−^, luciferase activity in promastigotes dropped to undetectable levels, while treated amastigotes had comparable luciferase activity to untreated controls.

### *L. amazonensis* encode a TXNPx isoform preferentially expressed by amastigotes

**Figure 3:**
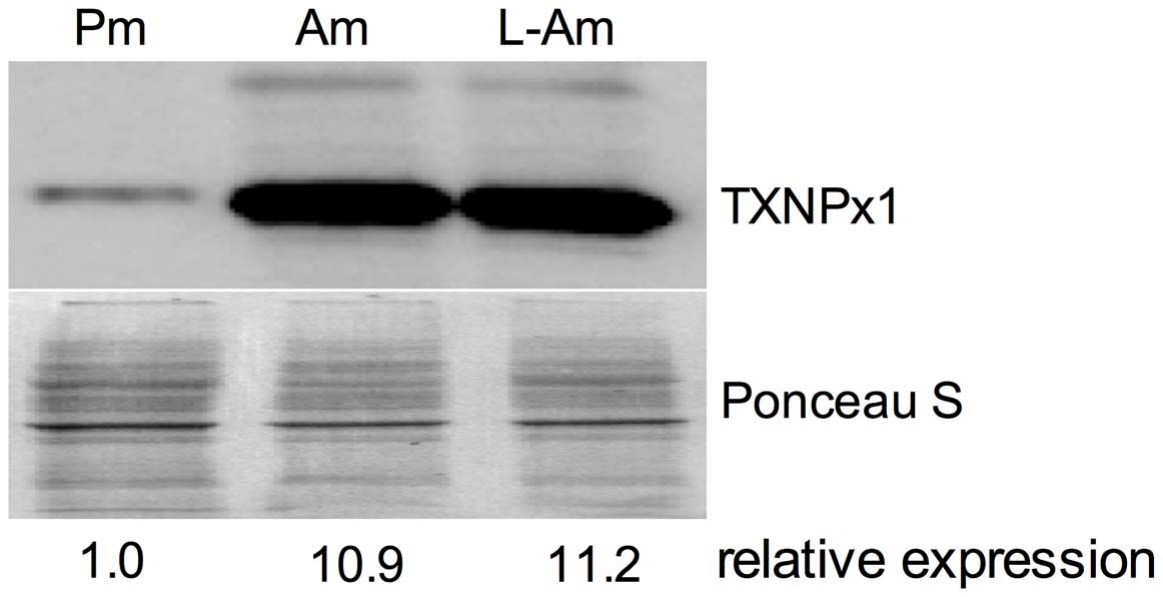
A unique tryparedoxin peroxidase (TXNPx1) isoform is highly expressed by mammalian stage amastigotes. TXNPx1 isoform protein levels in promastigotes (Pm), amastigotes (Am), and lesion-derived amastigotes (L-Am) determined by immunoblot. Relative density represents the mean band intensity from two independent immunoblots. Ponceau S staining of the membrane was used as a loading control.

The hyper-resistance of amastigotes to ONOO^−^ suggests that this form of the parasite may possess stage-specific antinitrosative defenses. A truncated TXNPx isoform capable of detoxifying both ROS and RNS and highly expressed by *L. chagasi* amastigotes was previously described [21]. Based on the enhanced resistance of amastigotes to RNS, we compared the expression level of the 190-amino acid truncated TXNPx1 isoform in *L. amazonensis* promastigotes and amastigotes. Immunoblots using an antibody against the C-terminal 40 amino acids of the *L. pifanoi* truncated TXNPx1 isoform [42] revealed that *L. amazonensis* axenic amastigotes expressed 11-fold higher levels of TXNPx1 than stationary-phase promastigotes (Figure 3). Moreover, expression of the TXNPx1 was also increased in lesion-derived amastigotes (Figure 3), indicating that this TXNPx isoform is highly expressed by parasites in developing cutaneous lesions.

### Intracellular TXNPx1 expression is induced in an iNOS-dependent manner

**Figure 4:**
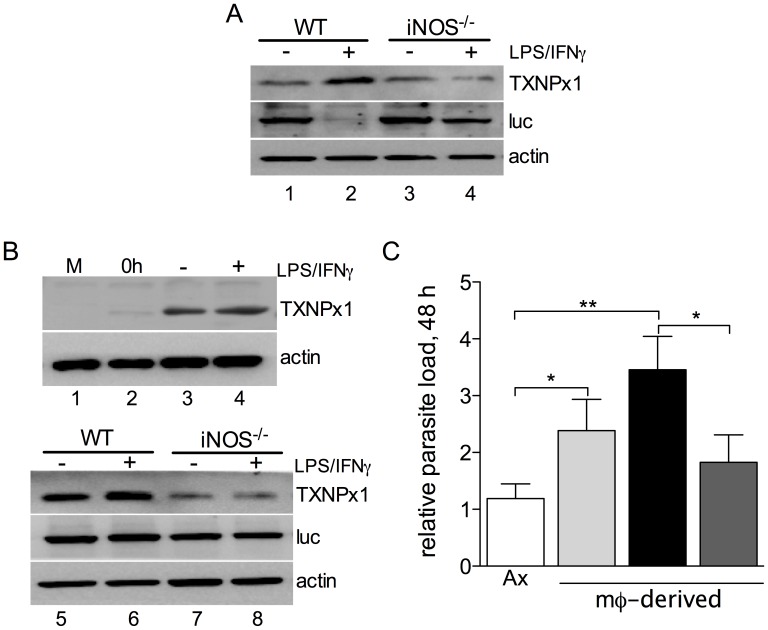
*L. amazonensis* TXNPx1 induction in infected MΦs is iNOS-dependent. A) Expression of TXNPx1 in control and IFN-γ/LPS-activated wild-type (WT) and iNOS^−/−^ MΦs at 4 days after infection with luciferase-expressing promastigotes (MOI 2). Host β-actin was used as a loading control and luciferase (luc) as an indicator of parasite loads. **B**) TXNPx1 expression in control and IFN-γ/LPS-activated WT and iNOS^−/−^ MΦs at 48 h post-infection with luciferase-expressing axenic amastigotes (MOI 2). Host β-actin was used as a loading control and luciferase (luc) as an indicator of parasite loads. C) Amastigotes were harvested 2 days post-infection from control MΦs (light grey bar) or cytokine-activated MΦs with (dark grey bar) or without (black bar) the iNOS inhibitor aminoguanidine, and the virulence of MΦ-derived amastigotes was compared to axenic amastigotes (Ax, white bar) in IFN-γ/LPS-activated MΦs. Parasite loads were determined at 48 h post-infection by using qPCR and calculated by the ΔΔC(t) method. * *p*<0.05, ** *p*<0.01.

Given that amastigotes have increased levels of TXNPx1, and that this antioxidant could mediate parasite protection against MΦ antimicrobial activity, we next determined TXNPx1 protein expression during the course of *Leishmania* infection in resting and cytokine-activated MΦs. Immunoblots from promastigote-infected MΦs 4 days post-infection indicated that parasite TXNPx1 expression was induced in cytokine-activated MΦs (Figure 4A, lanes 1 vs. 2). We found a reciprocal relationship between TXNPx1 expression levels and luciferase-expressing parasites in cytokine-activated MΦs, suggesting that conditions that negatively affect parasite survival also increased TXNPx1 expression (Figure 4A, lanes 1 vs. 2). Given the predicted role of TXNPx1 in RNS detoxification, we then evaluated whether TXNPx1 induction by intracellular parasites was dependent on a functional iNOS hemoprotein. Interestingly, the increase in TXNPx1 expression levels observed in activated MΦs was iNOS-dependent, as judged by the reduced TXNPx1 levels in activated iNOS^−/−^ cells compared to wild-type cells (Figure 4A, lanes 3 vs. 4). At 48 h post-infection, TXNPx1 expression was also increased in amastigote-infected wild-type cells (Figure 4B, lanes 2 vs. 3). Moreover, TXNPx1 expression was higher in IFN-γ/LPS-activated MΦs (Figure 4B, lanes 3 vs. 4 and 5 vs. 6). Similar to the findings for promastigote infection, we found that the increase in TXNPx1 expression in amastigote-infected control and activated MΦs was dependent on iNOS, as indicated by the limited TXNPx1 expression in iNOS^−/−^ cells (Figure 4B, lanes 7 vs. 8).

Due to the induction of TXNPx1 observed in response to host cell activation, we hypothesized that amastigotes derived from cytokine-activated MΦs (TXNPx1^high^) would possess a growth advantage over axenic amastigotes (TXNPx1^low^) in subsequent infection of activated MΦs. To test this hypothesis, we recovered amastigotes from primary cultures [control MΦ, and IFN-γ/LPS-activated MΦs (with or without the iNOS inhibitor aminoguanidine)] and then used them to infect IFN-γ/LPS-activated MΦs (secondary cultures). Cell-derived amastigotes exhibited a statistically significant increase in infectivity compared to axenic amastigotes at 48 h post-infection (Figure 4C, light grey bar). Importantly, TXNPx1^high^ amastigotes harvested from activated MΦs were highly infectious, showing a 3-fold higher parasite load at 48 h post-infection compared to TXNPx1^low^ axenic amastigotes (Figure 4C, black bar, *p*<0.01). Of note, this enhanced parasite fitness was diminished in the secondary infection if an iNOS inhibitor was included during primary infection (Figure 4C, dark grey bar). Therefore, the parasite response to MΦ RNS is crucial for fostering increased parasite fitness and infectivity. Collectively, these data show that parasites adapt to the harsh intracellular environment of MΦs by increasing antioxidant/antinitrosative defenses, including TXNPx1 induction, which promotes their infectivity and fitness in MΦs.

### Overexpression of the TXNPx1 isoform increases intracellular parasite survival *in vitro*, and parasite growth in a murine model of cutaneous leishmaniasis

**Figure 5:**
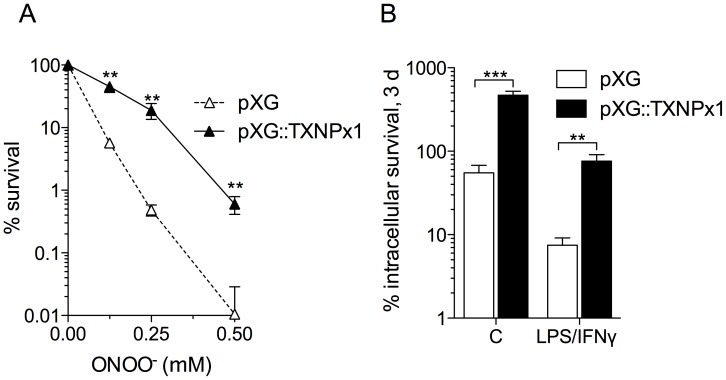
Overexpressing the 190-amino acid TXNPx1 isoform boosts promastigote resistance to ONOO^−^- and macrophage-mediated parasite killing. A) ONOO^−^ susceptibility and B) intracellular survival of promastigotes stably transformed with pXG (open symbols, bars) or pXG::TXNPx1 (black symbols, bars) in control and cytokine-activated macrophages. MΦs were activated with LPS (100 ng/mL) and IFN-γ (100 U/mL) for 16 h prior to infection. % survival was calculated by comparing the number of parasites in treated vs. untreated controls, or the parasite burden 3 days post-infection relative to the parasite burden after 1 h of internalization (t = 0). Infections were performed with an MOI of 2. The data represent the mean % survival ± SD of 3–5 independent observations from 2 separate experiments. ** *p*<0.01, *** *p*<0.001 compared to empty vector transformed controls.

**Figure 6:**
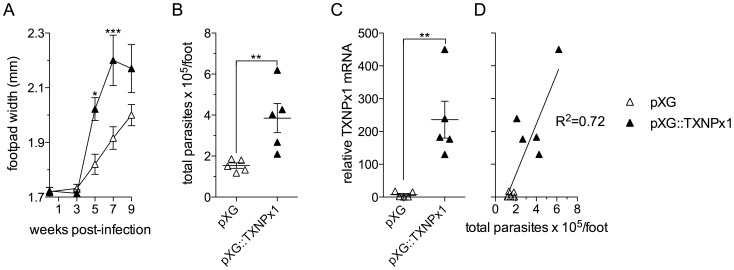
TXNPx1 expression fosters parasite growth and disease progression in a murine model of cutaneous leishmaniasis. C57BL/6 mice were inoculated s.c. in the right hind foot with 5×10^6^ stationary-phase promastigotes stably transformed with pXG (open symbols) or pXG::TXNPx1 (closed symbols). A) Lesion development was measured by biweekly caliper measurements. B) Parasite loads were determined by qRT-PCR based on the mRNA level of the constitutively transcribed *Ubiq* gene. C) *TXNPx1* expression levels in lesions were determined by qRT-PCR. Relative *TXNPx* mRNA was calculated by using *Ubiq* mRNA levels to normalize for parasite load, setting control levels to 1. D) Linear regression analysis of relative *TXNPx* mRNA and parasite load. The data correspond to the mean ± S.E.M. of data from 5 mice. * *p*<0.05, ** *p*<0.01, *** *p*<0.001 compared to empty vector controls.

To determine whether amastigotes' hyper-resistance to RNS was attributable to increased expression of TXNPx1, we generated an *L. amazonensis* promastigote strain that was stably transfected with the episomal pXG vector harboring the TXNPx1 open-reading frame. Transformed parasites had 3-fold higher TXNPx1 protein expression compared to control parasites carrying the empty pXG vector alone (Figure S3A). Both transformed parasite strains exhibited similar growth kinetics in complete Schneider's medium supplemented with 20% FBS, reaching stationary phase ∼5 days after passage at 1×10^5^ parasites/mL medium (Figure S3B). In addition, the yield of metacyclic promastigotes from both late stationary phase transformant cultures was similar (Figures S3C), indicating that TXNPx1 overexpression did not interfere with metacyclogenesis. Both parasite strains had similar sensitivity to H_2_O_2_ at concentrations between 0.125 and 2 mM (data not shown). In accordance with the proposed role of TXNPx1 in parasite resistance to nitroxidative stress and increased intracellular survival, promastigotes overexpressing the TXNPx1 isoform were 100-fold more resistant to ONOO^−^-mediated cytotoxicity than wild-type promastigotes after treatment with 250 µM ONOO^−^ (Figure 5A, closed symbols). Moreover, TXNPx1-overexpressing promastigotes exhibited enhanced intracellular survival in both resting and cytokine-activated MΦs (Figure 5B). Given the intra-MΦ survival advantage exhibited by the TXNPx1-overexpressing parasites, we hypothesized that these parasites could also have increased virulence in murine models. Accordingly, C57BL/6 mice infected with the TXNPx1-overexpressing promastigotes had enhanced lesion development compared to mice infected with control promastigotes, and this elevated pathogenicity correlated to an increase in parasite growth (Figure 6A and B). Using qRTPCR, we confirmed that TXNPx1 mRNA expression was 250-fold higher in cutaneous lesions from TXNPx1-overexpressing parasite-infected mice compared to control lesions (Figure 6C). There was a positive correlation between TXNPx1 mRNA levels and parasite load (Figure 6D, R^2^ = 0.72). Collectively, these data indicate that increased TXNPx1 expression confers resistance to macrophage microbicidal mechanisms and promotes *Leishmania*-mediated disease progression.

## Discussion

*Leishmania* persistence in the phagolysosome is indicative of the parasite's ability to counter host microbicidal activities. Since amastigotes excel in surviving and replicating within mononuclear phagocytes, it is to be expected that this stage possesses exclusive mechanisms to resist and subvert host cell immune responses. Indeed, several studies have shown that amastigotes are much more efficient at disrupting host cell signaling pathways and antimicrobial mechanisms compared to their promastigote counterparts, including blocking NADPH oxidase assembly and suppressing iNOS expression [14], [15]. The majority of studies have focused on the ability of *Leishmania* to prevent MΦ activation [43]; however, there is limited knowledge of how the parasites directly defend against antimicrobial products they may encounter within host macrophages. To address some of these issues, we focused our study on parasite interactions with cytokine pre-activated MΦs and present several important findings herein. First, *L. amazonensis* amastigotes not only resist MΦ microbicidal mechanisms, but also grow robustly in cytokine-activated, antimicrobial-producing MΦs while these cells readily kill promastigotes. Amastigotes are intrinsically more resistant than promastigotes to authentic RNS, implying that they possess developmental stage-enhanced antinitrosative defenses (Figures 1 and 2). Second, neither promastigotes nor amastigotes apparently interfere with iNOS transcription and NO synthesis in MΦs if the signaling cascades have already been activated prior to the infection (Figure S2). This view is consistent with the inability of *L. amazonensis* to block NO production in infected mice [44]. Third, the hyper-resistance of amastigotes to RNS-mediated toxicity is linked to the increased expression of a unique TXNPx isoform (Figure 3), which belongs to an antioxidant family of proteins known to detoxify several leishmanicidal molecules produced by professional phagocytes [21]–[23], [45]. We have provided evidence that the TXNPx1 isoform can be induced by host-derived signals during the course of infection (Figures 4). More importantly, overexpression of the TXNPx1 in promastigotes increases their resistance to direct RNS treatment and MΦ-mediated killing mechanisms during *in vitro* infection, and also fosters parasite survival and disease progression in an acute murine model of cutaneous leishmaniasis (Figures 5 and 6). Our findings support the notion that *L. amazonensis* amastigotes possess distinct resistance strategies that enable them to flourish in the intracellular environment of host cells, causing persistent infection and disease, even in hosts with Th1-like, proinflammatory responses.

As expected, promastigotes of *L. amazonensis* are highly efficient in establishing infection in resting MΦs *in vitro*, and are capable of replicating in these cells. It was noteworthy that only a small proportion of promastigotes survived the initial killing mechanisms in IFN-γ/LPS-activated, RNS-loaded MΦs, and that the resultant intracellular-transformed amastigote population started to expand around 2 days post-infection. Evidently, constitutively expressed antioxidant molecules in promastigote forms are insufficient to protect them against leishmanicidal molecules elaborated by activated MΦs. We, and others, have previously reported the failure to completely eliminate *L. amazonensis* promastigotes from classically-activated MΦs *in vitro*, which is in contrast to promastigotes of *L. major* and *L. braziliensis* [31], [46], [47]; this is presumably due to the relatively rapid induction of antioxidant capacity by *L. amazonensis* promastigotes in infected cells [30]. However, due to some potential caveats of using luciferase as a read-out for parasite viability, it is imperative that this assay be coupled with a second parasite quantification method (such as qRT-PCR) in order to make the most valid conclusions

Our findings of a positive correlation between growth of *L. amazonensis* amastigotes in cytokine-activated MΦs and expression of a developmentally regulated TXNPx1 are significant. The data indicate that the enhanced resistance of *L. amazonensis* amastigotes against MΦ antimicrobial activity is closely linked to a developmentally regulated factor capable of directly antagonizing host-derived antimicrobial products. Both *L. pifanoi* [42], and *L. amazonensis* (Henard *et al*., manuscript in preparation) express naturally truncated TXNPx1 isoforms (190 amino acid proteins) with high homology to the 199-amino acid cytosolic TXNPx except for the C-terminus, which presumably participates in the differential regulation of the TXNPx isoforms. *L. chagasi* (a causative agent of visceral leishmaniasis in the New World) and *L. aethiopica* (a causative agent of cutaneous leishmaniasis in the Old World) also express a truncated TXNPx1 isoform, also referred to as peroxidoxin 1 (Pxn1), that is preferentially expressed by the amastigote stage [21], [26]. Interestingly, the *L. chagasi* TXNPx1 (Genebank accession # AAG40074.1) detoxifies both ROS and RNS, while the function of its 199-amino acid TXNPx enzymes (Genebank accession # AAK69586.1 and AAK69587.1) is limited to ROS [22]. It is likely that the developmentally regulated TXNPx1 is not solely responsible for amastigote resistance to macrophage killing; rather, we suspect that it is part of a complex antioxidant armamentarium, resulting in enhanced resistance of amastigotes to macrophage microbicidal activity. A careful evaluation of the role of the TXNPx1 isoform *in vivo* will be benefited by the use of a TXNPx1 transgene with mutations in its redox active cysteine residues critical for antioxidant activity. At present, there are no reported studies identifying differentially regulated TXNPx1 isoforms in *L. major*, *L. donovani*, *L. infantum*, or *L. tropica*, even though their genome sequences are predicted to encode this truncated TXNPx isoform. Ongoing studies in our laboratory are focused on delineating the presence and regulation of other novel antioxidants in several Old and New World *Leishmania* species.

The marked amastigote resistance to host-derived antimicrobial molecules is likely critical for intracellular parasite survival in several different cell types during both acute and chronic phases of disease. In addition to MΦs, *Leishmania* are phagocytosed by polymorphonuclear neutrophils and dendritic cells at the site of infection [48], [49]. Indeed, we have recently showed that *L. amazonensis* amastigotes resist killing by murine neutrophils despite activating the respiratory burst in these cells [50]. Importantly, the enhanced antioxidant/antinitrosative defenses associated with *L. amazonensis* amastigotes likely enable the parasite to direct its resources to manipulate host cell function rather than repairing cellular damage caused by host-derived antimicrobials. Intracellular parasite killing is required for antigen presentation [51], [52], and leishmanial antigen presentation by dendritic cells and MΦs is critical for eliciting protective CD4^+^ T cell responses that control parasite burden and the disease. Therefore, it is not surprising that *L. amazonensis*-infected dendritic cells exhibit decreased activation and antigen presentation capabilities compared to *L. major*- and *L. braziliensis*-infected cells [53], [54], likely due to the superior host antimicrobial resistance of *L. amazonensis* compared to other *Leishmania* species [30]. In the future, it will be interesting to examine a correlation between the antioxidant capacity of intracellular parasites and their ability to modulate host cell functions.

In a broader context, the findings from this study have several important clinical implications. For example, IFN-γ/TNF-α and RNS production can be detected during chronic human leishmaniasis [55], [56], and the development of drug resistance in *Leishmania* is closely linked to the redox biology of the parasite. Several antimony-resistant *Leishmania* field isolates have had elevated trypanothione levels, and thiol depletion of these strains reestablishes antimony susceptibility [57]. Moreover, the overexpression of trypanothione-dependent TXN and TXNPx enzymes has been linked to antimony resistance in cutaneous and visceral leishmaniasis [25], [29], [58], and metastasis in mucocutaneous leishmaniasis [59]. Intriguingly, antimony-resistant parasites exhibit hyper-resistance to direct treatment with nitrogen oxides [60], and show enhanced intracellular survival within MΦs [25], [61]. At this stage, our understanding of the intracellular role of the TXNPx1 isoform remains incomplete. Specifically, it is unclear whether the TXNPx1 has additional functions independent of its antioxidant activity, or whether it plays a role in altering host gene expression. Nevertheless, a better understanding of *Leishmania* antioxidant and antinitrosative defenses may lead to the rational design of novel therapeutics for leishmaniasis.

In summary, our findings that *L. amazonensis* amastigotes are highly resistant to MΦ microbicidal defense and respond to NO congeners generated by iNOS are biologically important. Our results indicate that the amastigote-predominant TXNPx1 defends against the antimicrobial effects of ONOO^−^, and contributes to the unique resistance mechanisms that foster intracellular parasite survival and disease. Since *L. amazonensis* can cause progressive lesions in patients with diffuse cutaneous leishmaniasis and in most inbred strains of mice and exhibits increased resistance to iNOS-mediated killing compared to other *Leishmania* species [30], *L. amazonensis* is an excellent model for delineating antinitrosative defense mechanisms important for leishmaniasis and drug resistance.

## Supporting Information

Figure S1 ***L. amazonensis*** **amastigotes antagonize macrophage antimicrobial defenses.** Survival of luciferase-expressing promastigotes (Pm, A) and amastigotes (Am, B) in control MΦs (white symbols/bars) and IFN-γ/LPS-activated MΦs (black symbols/bars). MΦs were activated with LPS (100 ng/mL) and IFN-γ (100 U/mL) for 16 h prior to infection. C) Luciferase-expressing Pm and Am survival in control and IFN-γ/LPS-activated human THP-1 monocytes at 2 days post-infection. All infections were performed with an MOI of 2. The data represent the mean % survival ± SD of 4–8 independent observations from at least 2 separate experiments. *** *p*<0.001 compared to infected controls.
(TIFF)

Figure S2 **Promastigotes and amastigotes of** ***L. amazonensis*** **do not block iNOS activity in pre-activated MΦs.** Bone marrow-derived MΦs were activated with LPS (100 ng/mL) and 100 U/mL IFN-γ (100 U/mL) for 16 h, and then infected with luciferase-expressing promastigotes or amastigotes of *L. amazonensis* (MOI 2). Nitric oxide production at the time of infection (A) and at 2 days post-infection (B) was determined by measuring nitrite accumulation in the culture supernatants by using the Griess method. NO concentrations were calculated by regression analysis compared to a sodium nitrite standard. Data are presented as the mean ± SD of 3 independent observations obtained in 2 separate experiments. *** *p*<0.001 compared to unactivated controls.
(TIFF)

Figure S3 **Promastigotes overexpressing the 190-amino acid TXNPx1 isoform have similar growth kinetics and generation of metacyclic forms.** A) The levels of TXNPx1 protein in promastigotes stably transformed with pXG or pXG::TXNPx1. Relative density represents the mean ratio of TXNPx1 to the tubulin loading control from two independent immunoblots. B) Transformed parasite growth in complete Schneider's medium was measured daily by direct counting using a hemacytometer. C) Metacyclic forms from 7-day-old stationary-phase promastigote cultures grown in complete Schneider's medium were purified by using the 3A.1 mAb, and enumerated by direct counting using a hemacytometer. The data in B and C represent the mean parasites/mL ± SD of 3 independent observations.
(TIFF)

Figure S4 **Uncropped immunoblots.** A) Western blots depicted in Figure 3. M, molecular weight marker; lane 1, promastigote (Pm); lane 2, amastigote (Am); lane 3, lesion-derived amastigotes (L-Am). B) Western blots depicted in Figure 4A evaluating TXNPx1 expression in luciferase (luc)-expressing promastigote (Pm)-infected MΦs. Lane 1, control wild-type MΦs infected with Pm; lane 2, IFN-γ/LPS-activated wild-type MΦs infected with Pm; lane 3, control iNOS^−/−^ MΦs infected with Pm; lane 4, IFN-γ/LPS-activated iNOS^−/−^MΦs infected with Pm. All lysates were generated 3 d post-infection C) Western blots depicted in Figure 4B evaluating TXNPx1 expression in amastigote (Am)-infected MΦs. Lane 1, mock-infected MΦs; lane 2, control wild-type MΦs infected with Am at 0 d post-infection; lanes 3 and 5, control wild-type MΦs infected with Am 2 d post-infection; lanes 4 and 6, IFN-γ/LPS-activated wild-type MΦs infected with Am 2 d post-infection; lane 7, control iNOS^−/−^ MΦs infected with Am 2 d post-infection; lane 8, IFN-γ/LPS-activated iNOS^−/−^ MΦs infected with Am 2 d post-infection.
(TIFF)
