# Supplementary figures and images for: Leishmania amazonensis Amastigotes Highly Express a Tryparedoxin Peroxidase Isoform That Increases Parasite Resistance to Macrophage Antimicrobial Defenses and Fosters Parasite Virulence
Source: PLoS Negl Trop Dis. 2014 Jul 17;8(7):e3000. doi: 10.1371/journal.pntd.0003000 (PMC4102420; doi:10.1371/journal.pntd.0003000)

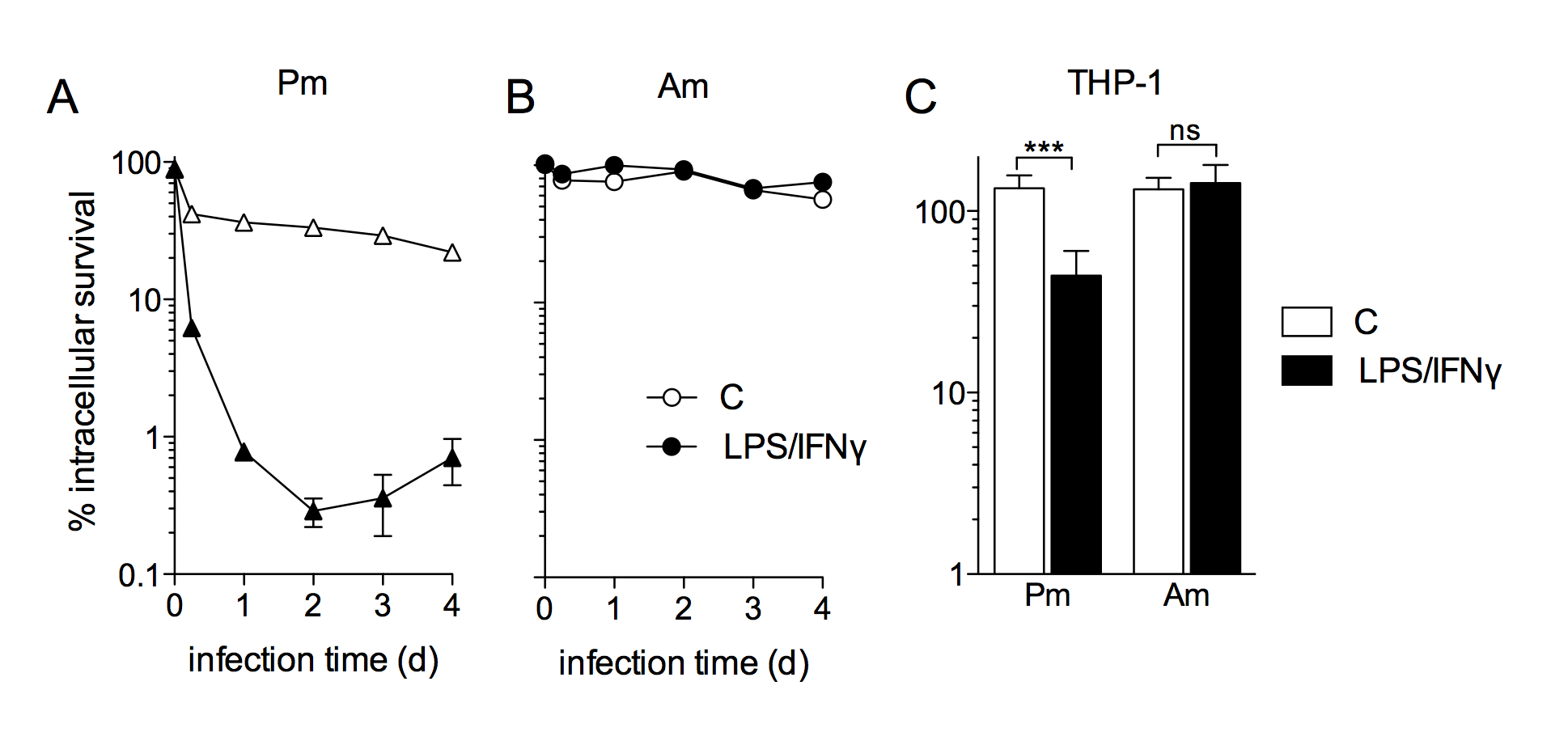

Supplement: Figure S1 — L. amazonensis amastigotes antagonize macrophage antimicrobial defenses. Survival of luciferase-expressing promastigotes (Pm, A) and amastigotes (Am, B) in control MΦs (white symbols/bars) and IFN-γ/LPS-activated MΦs (black symbols/bars). MΦs were activated with LPS (100 ng/mL) and IFN-γ (100 U/mL) for 16 h prior to infection. C) Luciferase-expressing Pm and Am survival in control and IFN-γ/LPS-activated human THP-1 monocytes at 2 days post-infection. All infections were performed with an MOI of 2. The data represent the mean % survival ± SD of 4–8 independent observations from at least 2 separate experiments. *** p<0.001 compared to infected controls. (TIFF) [file pntd.0003000.s001.tiff]

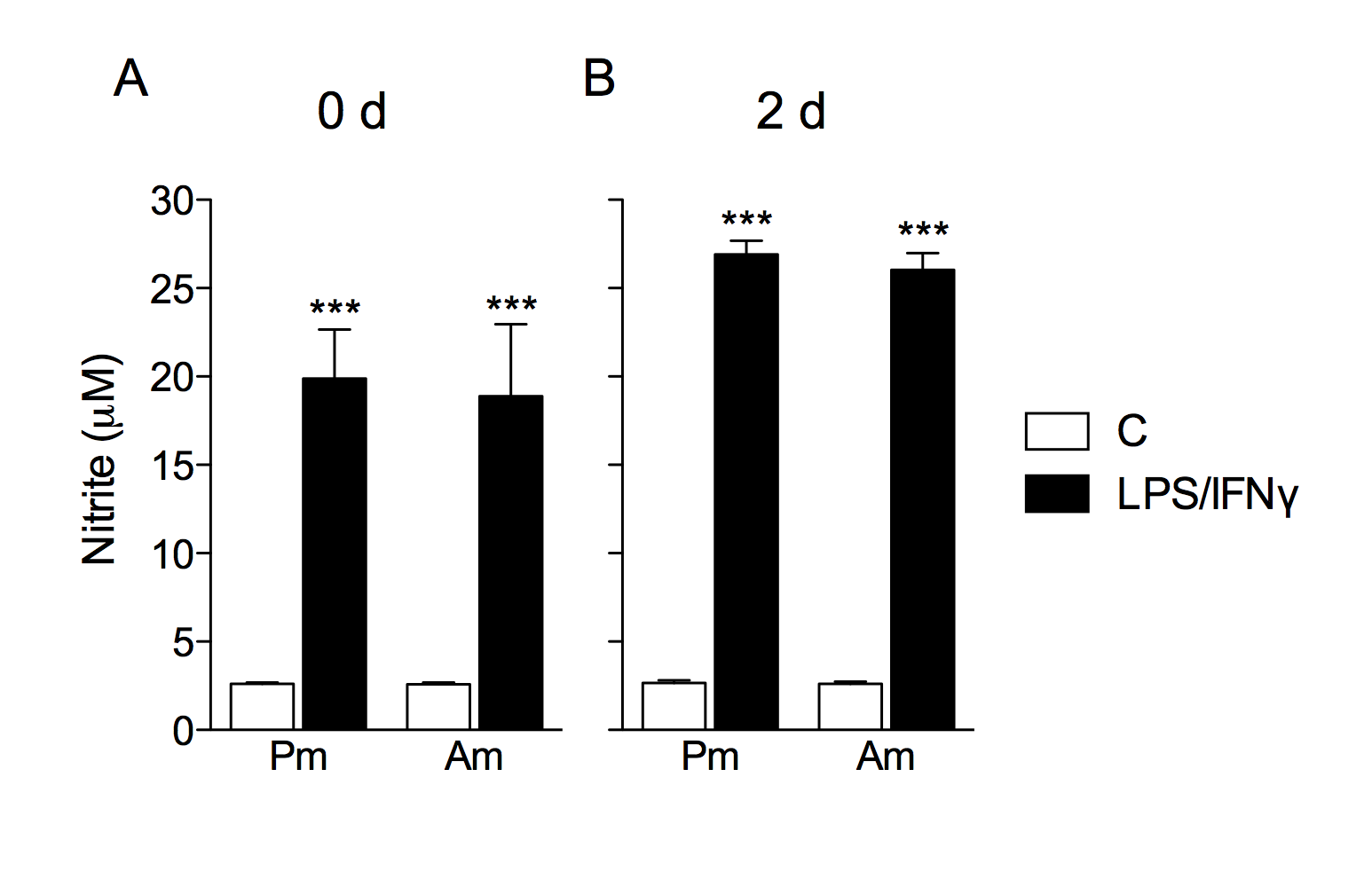

Supplement: Figure S2 — Promastigotes and amastigotes of L. amazonensis do not block iNOS activity in pre-activated MΦs. Bone marrow-derived MΦs were activated with LPS (100 ng/mL) and 100 U/mL IFN-γ (100 U/mL) for 16 h, and then infected with luciferase-expressing promastigotes or amastigotes of L. amazonensis (MOI 2). Nitric oxide production at the time of infection (A) and at 2 days post-infection (B) was determined by measuring nitrite accumulation in the culture supernatants by using the Griess method. NO concentrations were calculated by regression analysis compared to a sodium nitrite standard. Data are presented as the mean ± SD of 3 independent observations obtained in 2 separate experiments. *** p<0.001 compared to unactivated controls. (TIFF) [file pntd.0003000.s002.tiff]

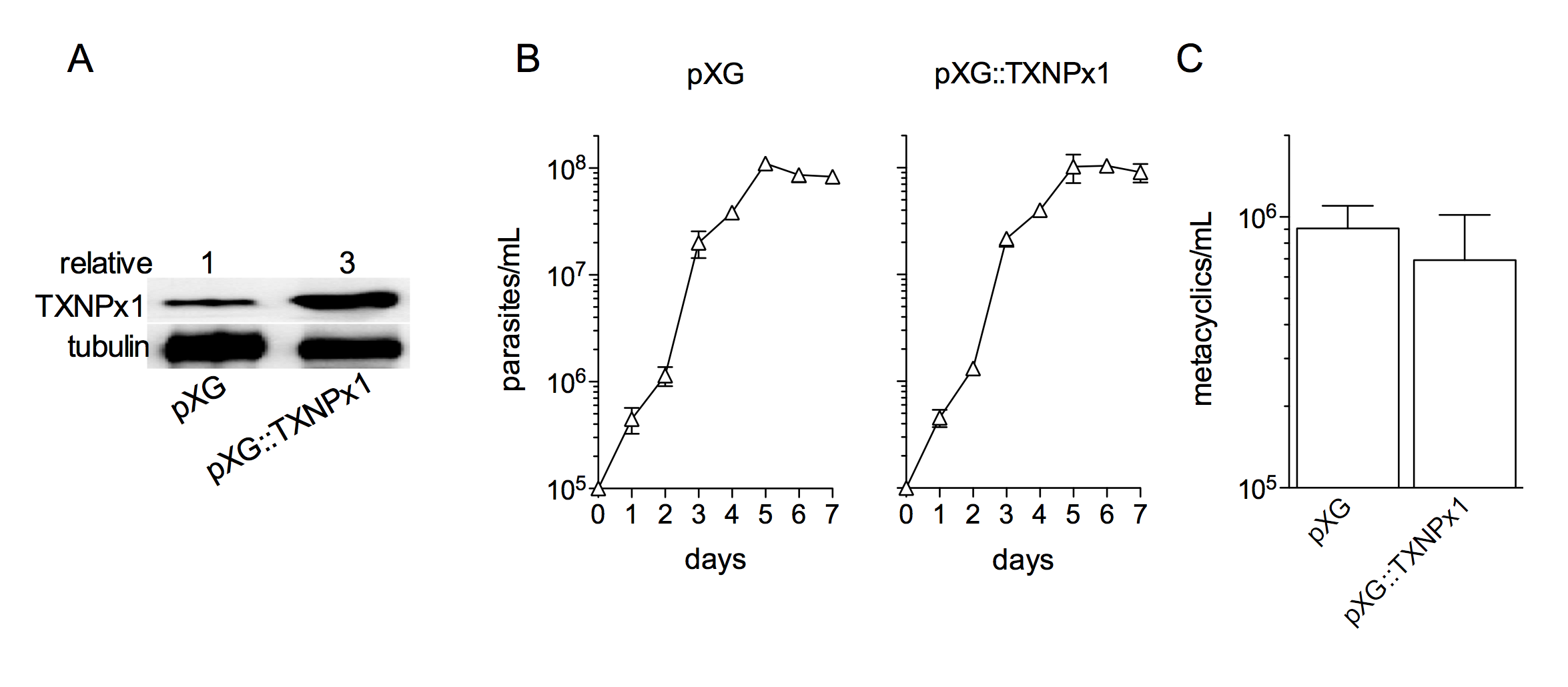

Supplement: Figure S3 — Promastigotes overexpressing the 190-amino acid TXNPx1 isoform have similar growth kinetics and generation of metacyclic forms. A) The levels of TXNPx1 protein in promastigotes stably transformed with pXG or pXG::TXNPx1. Relative density represents the mean ratio of TXNPx1 to the tubulin loading control from two independent immunoblots. B) Transformed parasite growth in complete Schneider's medium was measured daily by direct counting using a hemacytometer. C) Metacyclic forms from 7-day-old stationary-phase promastigote cultures grown in complete Schneider's medium were purified by using the 3A.1 mAb, and enumerated by direct counting using a hemacytometer. The data in B and C represent the mean parasites/mL ± SD of 3 independent observations. (TIFF) [file pntd.0003000.s003.tiff]

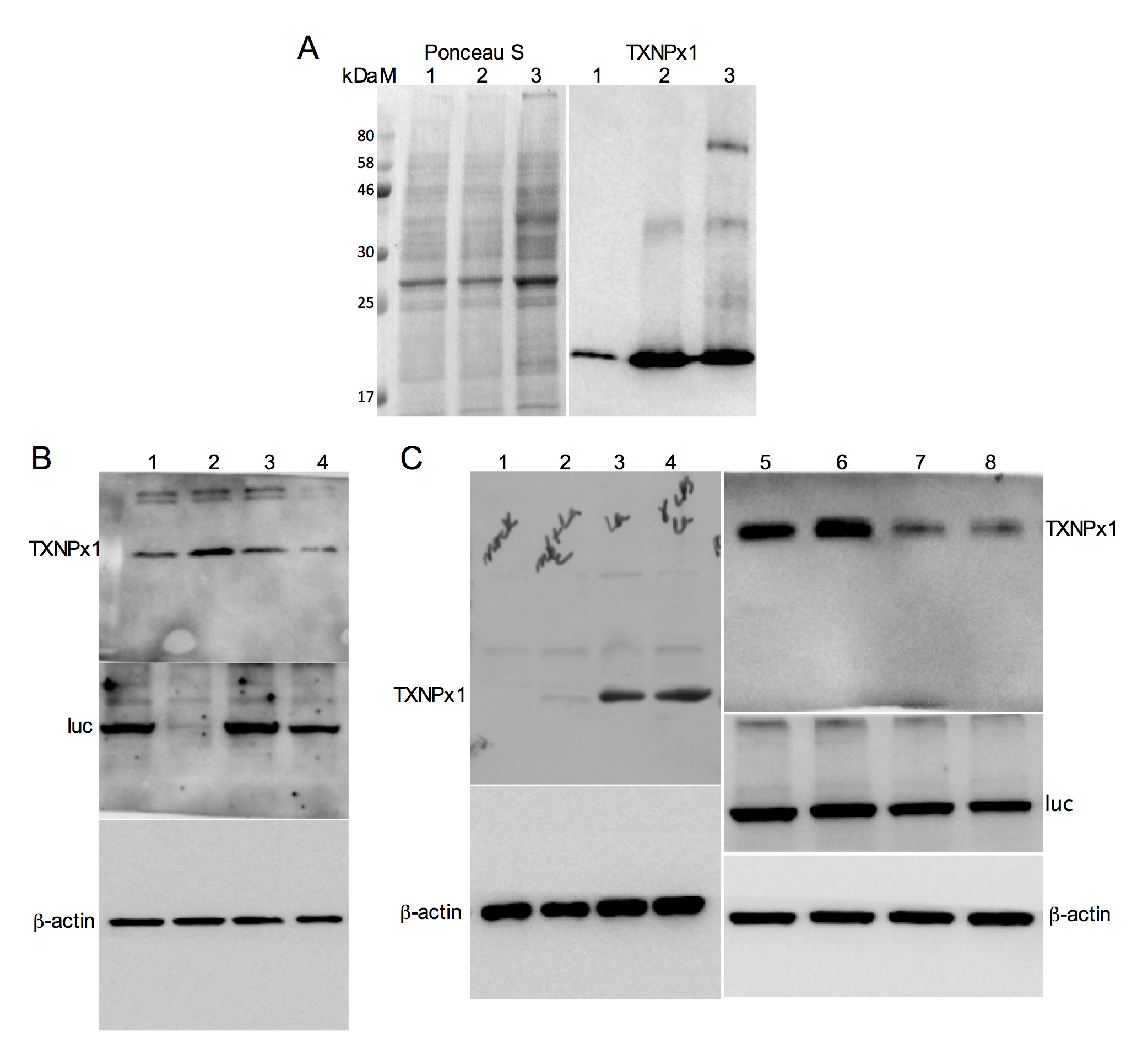

Supplement: Figure S4 — Uncropped immunoblots. A) Western blots depicted in Figure 3. M, molecular weight marker; lane 1, promastigote (Pm); lane 2, amastigote (Am); lane 3, lesion-derived amastigotes (L-Am). B) Western blots depicted in Figure 4A evaluating TXNPx1 expression in luciferase (luc)-expressing promastigote (Pm)-infected MΦs. Lane 1, control wild-type MΦs infected with Pm; lane 2, IFN-γ/LPS-activated wild-type MΦs infected with Pm; lane 3, control iNOS−/− MΦs infected with Pm; lane 4, IFN-γ/LPS-activated iNOS−/−MΦs infected with Pm. All lysates were generated 3 d post-infection C) Western blots depicted in Figure 4B evaluating TXNPx1 expression in amastigote (Am)-infected MΦs. Lane 1, mock-infected MΦs; lane 2, control wild-type MΦs infected with Am at 0 d post-infection; lanes 3 and 5, control wild-type MΦs infected with Am 2 d post-infection; lanes 4 and 6, IFN-γ/LPS-activated wild-type MΦs infected with Am 2 d post-infection; lane 7, control iNOS−/− MΦs infected with Am 2 d post-infection; lane 8, IFN-γ/LPS-activated iNOS−/− MΦs infected with Am 2 d post-infection. (TIFF) [file pntd.0003000.s004.tiff]
